# Resveratrol inhibits androgen production of human adrenocortical H295R cells by lowering CYP17 and CYP21 expression and activities

**DOI:** 10.1371/journal.pone.0174224

**Published:** 2017-03-21

**Authors:** Nesa Marti, Nadia Bouchoucha, Kay-Sara Sauter, Christa E. Flück

**Affiliations:** 1 Division of Pediatric Endocrinology, Department of Pediatrics and Department of Clinical Research, University of Bern, Bern, Switzerland; 2 Graduate School of Bern, University of Bern, Bern, Switzerland; University of South Alabama Mitchell Cancer Institute, UNITED STATES

## Abstract

Resveratrol, a natural compound found in grapes, became very popular for its suggested protective effects against aging. It was reported to have similar positive effects on the human metabolism as caloric restriction. Recently, positive effects of resveratrol on steroid biosynthesis in cell systems and in humans suffering from polycystic ovary syndrome have also been reported, but the exact mechanism of this action remains unknown. Sirtuins seem targeted by resveratrol to mediate its action on energy homeostasis. In this study, we investigated the mechanisms of action of resveratrol on steroidogenesis in human adrenal H295R cells. Resveratrol was found to inhibit protein expression and enzyme activities of CYP17 and CYP21. It did not alter CYP17 and CYP21 mRNA expression, nor protein degradation. Only SIRT3 mRNA expression was found to be altered by resveratrol, but SIRT1, 3 and 5 overexpression did not result in a change in the steroid profile of H295R cells, indicating that resveratrol may not engage sirtuins to modulate steroid production. Previous studies showed that starvation leads to a hyperandrogenic steroid profile in H295R cells through inhibition of PKB/Akt signaling, and that resveratrol inhibits steroidogenesis of rat ovarian theca cells via the PKB/Akt pathway. Therefore, the effect of resveratrol on PKB/Akt signaling was tested in H295R cells and was found to be decreased under starvation growth conditions, but not under normal growth conditions. Overall, these properties of action together with recent clinical findings make resveratrol a candidate for the treatment of hyperandrogenic disorders such as PCOS.

## Introduction

Resveratrol (trans-3,5,4’-trihydroxysilbene; RSV) is a polyphenol, which is found in several plant species. Recently, it became very popular in the scientific community because of its suggested protective properties against age-related complications like metabolic disorders, cardio-vascular diseases and cancers [1–3]. Its ability to expand lifespan was shown in several organisms [4, 5] and resembles the known beneficial effect of caloric restriction (CR). CR enhances maximum lifespan in lower eukaryotes as well as mammals [6, 7], prevents age-related metabolic and cardiovascular effects, and lowers cancer incidence [8, 9]. Most recently, RSV has also been shown to lower androgen levels in women with polycystic ovary syndrome (PCOS), the most common reproductive disorder in women characterized by hyperandrogenism [10]. Not only that CR and RSV have many overlapping beneficial health effects, the transcriptome of key metabolic tissues from RSV treated mice is similar to the CR induced profile [11].

Starving human adrenocortical cells leads to an induction of steroidogenesis. Key enzymes of steroid biosynthesis are altered under starvation growth conditions such that enhanced CYP17 activity and impaired HSD3B2 activity lead to an increase in androgen production [12]. RSV, on the contrary, reduces androgen production in rat ovarian theca cells [13], and suppresses steroidogenesis in rat Leydig [14] and rat adrenocortical cells [15]. Moreover, RSV inhibits the activity of CYP17 in H295R cells [16]. Thus, while starvation and RSV show similar effects on some metabolic pathways in several biological systems, they seem to regulate steroidogenesis in the opposite way.

Another popular compound in the context of mimicking CR is metformin, which is known as first-line diabetes medication. This biguanid shows beneficial effects on general health and lifespan in different models [17, 18] and is used for the treatment of PCOS because of its antidiabetic and androgen-lowering properties. Human adrenocortical NCI-H295R cells grown under metformin treatment show lower CYP17-lyase as well as lower HSD3B2 activities [19]. Taken together, steroid biosynthesis of human adrenocortical cells is altered by starvation and by RSV or metformin treatment, respectively. The modulation of androgen production seems to target CYP17 activity, but the exact mechanism of action of RSV on CYP17 and overall steroidogenesis remains unclear.

On the other hand, the lifespan prolonging mechanism of RSV seems to involve sirtuins, a conserved family of NAD+ -dependent (class III) histone and protein deacetylases [4, 5]. The first identified member of the family, Sir2 (silent information regulator 2), was found to increase the yeast replicative lifespan by 30% [20]. In mammals there are seven orthologues to Sir2 (SIRT1-SIRT7), of which SIRT1 is the most extensively studied member and a known target of RSV. In rat ovarian granulosa cells, RSV was found to promote SIRT1 and StAR expression [21], and in human adrenocortical cells, RSV stimulated cortisol biosynthesis in a SIRT3- and SIRT5 -dependent manner [22].

In previous work, we showed that metformin inhibits the activity of complex I of the respiratory chain in H295R cells, thereby diminishing the NAD+ content in the cells [19](Hirsch et al., 2012). This finding suggests a possible effect of metformin on the NAD+ -dependent deacetylases, including the sirtuins. Moreover, different studies showed an effect of metformin on SIRT1 in several models including human granulosa cells [23]. Therefore, this knowledge led us to hypothesize that RSV as well as metformin may lower androgen production in human adrenocortical H295R cells via a sirtuin-dependent mechanism.

This paper aims at investigating RSVs possible mechanisms of action on human adrenal steroidogenesis overall, and on androgen production specifically. Using the H295R cell model for our studies, we found that resveratrol inhibits the activities of CYP17 and CYP21 without involving SIRT1, 3 or 5. RSV lowered protein, but not gene expression of CYP17 and CYP21. PKB/Akt signaling seemed involved.

## Materials and methods

Resveratrol was purchased from SIGMA (Saint Louis, Missouri, USA). Radioactive labeled [7(N)-^3^H]-pregnenolone (12.6 Ci/mmol) and [1,2,6,7(N)-^3^H]–DHEA (63 Ci/mmol) were obtained from PerkinElmer (Waltham, MA, USA). [4-^14^C]-progesterone (ART-1398) was purchased from American Radiolabeled Chemicals (St. Louis, MO, USA). Antibodies against human CYP17A1 and POR were custom made by GenScript (Piscataway, NJ, USA). Anti-CYP21A2 antibody was a generous gift of Prof. Walter L. Miller (San Francisco UCSF, CA, USA). The phospho-Akt pathway was studied using anti-Akt and anti-phospho-Akt (Ser 473) antibodies from Cell Signaling Technology (Danvers, MA, USA). Antibodies against SIRT1 (Abcam, Cambridge, UK), SIRT3 and SIRT5 (Cell Signaling, Danvers, MA, USA) were kindly shared by Dr. Jean-Marc Nouffer (University of Bern, Bern, Switzerland). β-actin antibody was obtained from Sigma-Aldrich (St. Louis, MO, USA). SIRT3/5 plasmids were obtained from the laboratory of Dr. Eric Verdin (University of California San Francisco, San Francisco, CA) via Addgene (Cambridge, MA) and provided by Prof Marion B. Sewer (UC San Diego, CA, USA). SIRT1 plasmid was obtained from Prof. Anna Biason-Lauber (University of Fribourg, Fribourg, Switzerland).

### NCI-H295R cell cultures

Human adrenal NCI-H295R cells purchased from American Type Culture Collection (ATCC; CRL-2128) were maintained under normal growth conditions (growth medium, GM) in DMEM/Ham’s F-12 medium containing L-glutamine and 15mM HEPES medium (GIBCO, Paisley, UK) supplemented with 5% NU-I serum (Becton Dickinson, Franklin Lakes, NJ USA), 0,1% insulin, transferrin, and selenium (100U/ml; GIBCO), 1% penicillin (100 U/ml; GIBCO) and streptomycin (100μg/ml; GIBCO). The serum-free NCI-H295R medium (starvation medium, SM) contained DMEM/Ham’s F-12 medium, penicillin (100 U/ml; GIBCO), and streptomycin (100 μg/ml; GIBCO) only. The cultures were kept at 37°C with 5% CO_2,_ and the cells were divided once a week. For steroid profiling, mRNA expression and protein expression experiments, cells with passage numbers 18 to 23 were subcultured for 24 hours in GM on 6-well plates and then starved, or alternatively treated with metformin and resveratrol. Metformin was dissolved in water and used at a final concentration of 1mM. Resveratrol was dissolved in DMSO and used at final concentrations of 5μM to 50μM.

### Labeling of steroidogenesis for measuring enzymatic conversions

Steroid metabolism was labeled by adding either 54‘000 cpm [^3^H]-pregnenolone, 49,000 cpm [^3^H]-DHEA or 59,000 cpm [^14^C]-progesterone per well for 120, 90 and 60 minutes, respectively. Steroids were extracted from medium as previously described [12] and separated on thin-layer chromatography (TLC) plates (Macherey-Nagel, Düren, Germany) using the chloroform:ethylacetate (3:1) solvent system. The separated steroids were visualized by exposing TLC plates on imaging screens and reading them on a Fuji PhosphoImager FLA-7000 (Fujifilm, Dielsdorf, Germany). Steroids were identified by running known standards in parallel on screens. Specific steroids were densitometrically quantified with the Multi Gauge software (Fujifilm). Finally, specific steroid conversion was calculated as a percentage of radioactivity incorporated in a specific steroid hot spot when compared with the total radioactivity added to the reaction.

### RNA isolation and quantitative Real Time PCR (qRT-PCR)

H295R cells were cultured under GM and SM conditions, and with resveratrol and metformin treatments. Total RNA was isolated using the TRIzol method according to the manufacturer's instructions (Invitrogen Life Technologies, Carlsbad, CA, USA). cDNA was produced using the Improm RNA transcriptase kit (Promega). qRT-PCR analysis was performed on the 7500-Fast real-time PCR System (Applied Biosystems, Foster City, CA, USA) using ABsolute SYBR Green Mix (ABgene; Thermo Fisher scientific, Waltham, MA, USA), specific primers (Microsynth, Balgach, Switzerland) and 50ng mRNA in a total volume of 25μl. Specific primers were newly designed using the NCBI primer designing tool Primer-Blast (https://www.ncbi.nlm.nih.gov/tools/primer-blast/). Primer sequences are given in Supplemental Material (S1 Table). Cycling conditions comprised a first incubation at 50°C for 2 min and a second incubation at 95°C for 10 min, followed by 40 cycles of 95°C for 15 sec and 60°C for 60 sec. *GAPDH* was used as endogenous control. Fold change in gene expression for a particular gene was calculated by the 2^−ΔΔCt^ method. Amplification curves and the mean cycle threshold (Ct) values were calculated using the 7500 Fast System SDS software (Applied Biosystems), and correction for the endogenous genes, ΔCt and ΔΔCt were calculated [19]

### Overexpression of sirtuins

H295R cells were transfected with cDNA expressing vectors for *hSIRT1*, *hSIRT3*, *hSIRT5* or with the empty vector pcDNA3. Transient transfection was carried out in 12-well plates for 6 h (Falcon 3047; Becton Dickinson) using Lipofectamin 2000 reagent (Invitrogen) according to the manufacturer’s recommendations. The transfection was performed in DMEM/Ham’s F-12 medium containing L-glutamine and 15mM HEPES medium (GIBCO, Paisley, UK) supplemented with 5% NU-I serum (Becton Dickinson, Franklin Lakes, NJ USA) and 0,1% insulin, transferrin, and selenium (100U/ml; GIBCO). After 6 hours of transfection, the transfection medium was replaced by GM for 24 hours. In the following 24 hours the cells were either grown under GM or SM conditions, or under RSV treatment. Before closing the experiment 48 hours after transfection, steroidogenesis was labeled by adding [^3^H]-pregnenolone for 90min. Supernatants were then collected to assess the steroid profile by TLC, and cells were washed with phosphate buffered saline (PBS) for Western blot analysis.

### Western blot analysis

Protein extraction and concentration measurements from cell cultures were performed as previously described [24]. Briefly, cells were harvested in lysis buffer, the lysates were pressed trough 25-gauge needles, centrifuged and then the supernatants were collected. Protein concentrations were measured using the Bio-Rad protein assay (Bio Rad Laboratories, Munich, Germany). Protein extracts were resolved in a SDS loading buffer (62.5 mM Tris-HCL, pH 6.8; 2% sodium dodecylsulfate, 10% glycerol, 100mM dithiotreitol, 0.01% bromophenol blue) and held at 95°C for 5 min. Samples, loaded in equal protein amounts, were separated on ExpressPlus PAGE gels (GenScript) and transferred by the semi-dry method on Immobilon-FL PVDF transfer membranes (Milipore, Billerica, MA, USA). Blocking was performed for 1 hour with 5% non-fat dry milk. Blots were incubated overnight with the primary antibodies under the following specific conditions: CYP17A1, CYP21A2, POR 1:5’000 in 4% milk/TTBS; SIRT1 1:1’700 in TTBs; SIRT3, SIRT5 1:1’250 in TTBS, β-actin 1:5’000 in 4%BSA/TTBS. Secondary antibodies [IRDye 800CW donkey anti-rabbit, IRDye 800CW donkey anti-chicken and IRDye 680 goat anti-mouse (LI-COR Bioscience Inc., Lincoln, NE, USA)] were used at a concentration of 1:15’000 in 5% milk/TTBS. Incubation time for the secondary antibodies was 1 hour at room temperature. Readout was done by scanning the membrane and quantifying the reactive band using the OdysseySA Infrared Image system (LI-COR Bioscience Inc.). Figures in the manuscript show representative captures of WB gels. All original WB are given as Supplemental Material (S1 Fig).

### Statistics

Data are the mean ± SD of three or more independent experiments. For qRT-PCR triplicates were performed in single experiments. Statistical analysis was performed using the two-tailed Student’s t-test when comparing two samples; group analysis was performed by ANOVA (Excel; Microsoft Corp., Redmond, WA, USA).

## Results

### Resveratrol inhibits androgen production in human adrenal H295R cells

**Fig 1:**
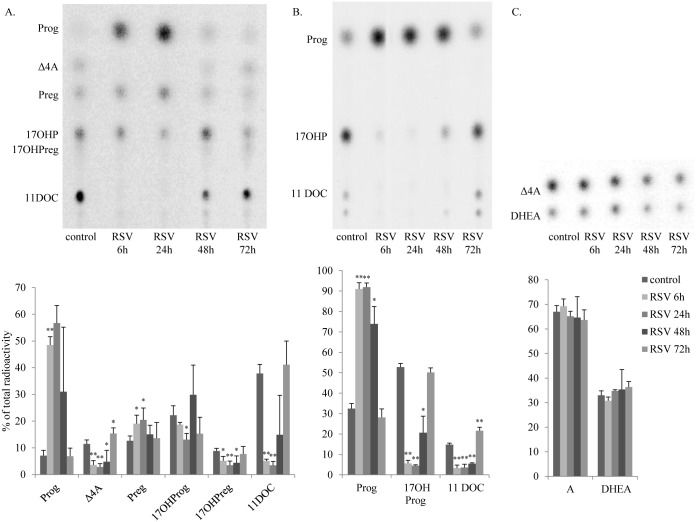
Resveratrol inhibits steroidogenesis in H295R cells. Steroid profiles of H295R cells treated for 6, 24 and 72 hours with 5μM resveratrol are shown. Steroid production was labeled with radioactive precursors and steroids were extracted and resolved by TLC. Representative TLCs are shown in the upper panel and quantitative analysis it shown in the graph in the lower panel. A. Overall, resveratrol impaired steroid production, e.g. reduced conversion of labeled [^3^H]-Preg to 17OHPreg, 17OHP, 11DOC and DHEA after 6 to 24 hours. B. Specifically, the conversion of [^14^C]-Prog to 17OHP and 11DOC catalyzed by the enzymes CYP17 and CYP21 were inhibited after resveratrol treatment. C. Resveratrol treatment did not affect the conversion of [^3^H]-DHEA to Δ4A by the enzyme HSD3B2. Data are expressed as the mean ± SD of four independent experiments. Test for statistical significance was done between two datapoints comparing each single treatment duration to the respective control,and was marked as * p<0.05, ** p<0.01. Calculations for group comparison by ANOVA confirmed significant changes. TLC, thin layer chromatography; RSV, resveratrol; Prog, progesterone; Δ4A, androstenedione; Preg, pregnenolone; 17OHPreg, 17-hydroxypregnenolone; 17OHP, 17-hydroxyprogesterone, 11DOC, 11-deoxycortisol.

To assess the effect of resveratrol on steroidogenesis in H295R cells, we performed steroid profiling of cell culture supernatants after incubating cells with different steroid substrates. Steroid profiling using radiolabeled pregnenolone (Fig 1A) showed lower conversion of progesterone to 17OH-progesterone, as well as a lower conversion of pregnenolone to 17OH-pregnenolone under resveratrol treatment when compared to untreated cells, indicating an inhibition of resveratrol on the CYP17-hydroxylase activity. Further, we found impaired androstenedione production, suggesting a possible inhibition of CYP17-lyase and HSD3B2 activities. Using labeled progesterone as precursor (Fig 1B), we confirmed the inhibition of CYP17-hydroxylase activity and also found an inhibited CYP21 activity, evidenced by lower conversion of progesterone to 17OH-progesterone and 11-deoxycorticosterone after 6 and 24 hours of resveratrol treatment (Fig 1B). By contrast, resveratrol treatment did not affect the conversion of DHEA to androstenedione, suggesting no effect on HSD3B2 activity (Fig 1C). The maximum effect of resveratrol on steroidogenesis in H295R cells occurred between 6 and 24 hours, and the effect of resveratrol vanished after 72 hours, indicating that the used resveratrol treatment of 5 μM was non-toxic for the cells.

### Resveratrol does not alter CYP17A1 and CYP21A2 activity by lowering gene expression

**Fig 2:**
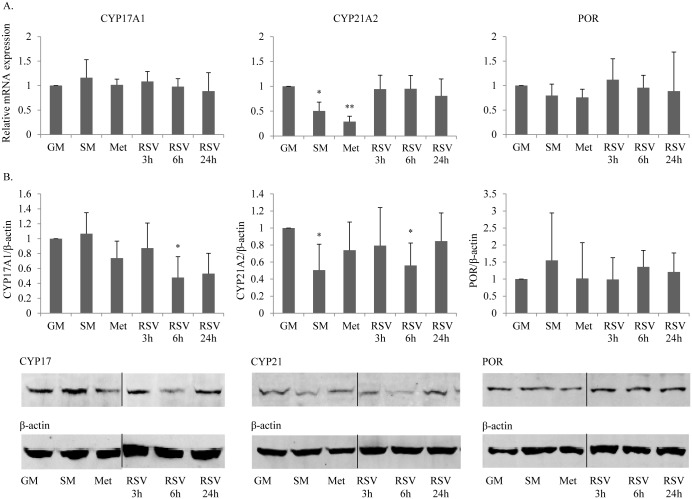
Resveratrol decreases CYP17A1 and CYP21A2 protein expression. A. Gene expression profiling by qRT-PCR was performed for CYP17, CYP21 and their cofactor POR in H295R cells grown under normal conditions (GM), starvation conditions (SM), metformin (Met) (1mM) treatment and under resveratrol (RSV) (5μM) treatment. None of the conditions or treatments affected RNA expression of CYP17, CYP21 or POR. Data are the mean ± SD of three independent SYBR Green based qRT-PCR experiments. B. Western blot analyses for CYP17, CYP21 and POR showed decreased CYP17 and CYP21 protein expression after 6 hours of resveratrol treatment. Data are the mean ± SD of four independent experiments (upper panel). Representative blots are shown in the lower panel. β-actin staining was used as loading control. * p<0.05.

To find the underlying mechanism of action how resveratrol affects CYP17 and CYP21 activities, we investigated the potential effect on gene expression of these enzymes. H295R cells were treated with resveratrol for 3 to 24 hours. For comparison, we also assessed gene expression under starvation growth conditions, a condition known to enhance androgen production [25], as well as under metformin treatment, known to impair androgen production [19, 24]. qRT-PCR using specific primers revealed no changes in the expression of *CYP21* after resveratrol treatment, but lower expression after starvation and metformin treatment (Fig 2A). *CYP17* gene expression was not affected by resveratrol, nor by starvation and metformin treatment (Fig 2A). These results indicated that resveratrol does not affect *CYP17* and *CYP21* gene transcription and translation, or RNA stability. Knowing that both CYP17 and CYP21 activities depend on cofactor cytochrome P450 oxidoreductase (POR) [26], we also assessed a possible effect of resveratrol on *POR* gene expression (Fig 2A). However, resveratrol was not found to change *POR* expression in H295R cells.

### Resveratrol lowers CYP17 and CYP21 protein expression

Changes of CYP17 and CYP21 activities were already observed after 3 hours of resveratrol treatment, with strongest effects after 6 hours (Fig 1); this pointed to a rather rapid regulation at the posttranscriptional level. Consistent with that, Western blot experiments using specific antibodies revealed significantly lower CYP17 and CYP21 protein expression after 6 hours of resveratrol treatment (Fig 2B). Protein expression of POR remained unchanged under resveratrol treatment (Fig 2B). In addition, CYP21 protein expression was lower in starved H295R cells and even lower after metformin treatment as previously shown [27].

### SIRT3 gene expression is up-regulated in H295R cells after resveratrol treatment

**Fig 3:**
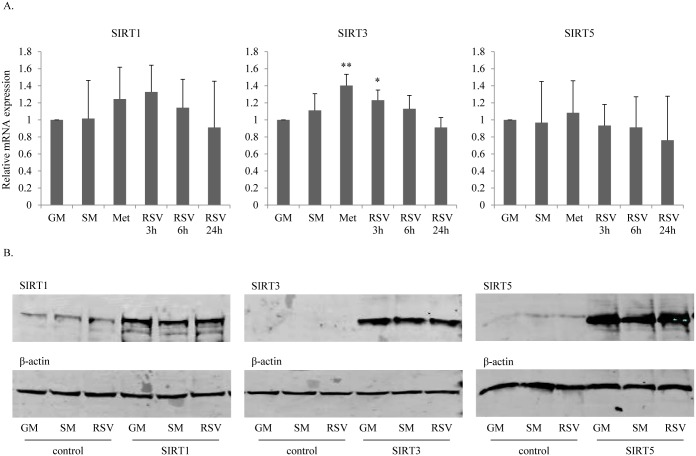
Resveratrol enhances SIRT3 RNA expression in H295R cells. A. qRT-PCR gene expression profiles were assessed for SIRT1, SIRT3 and SIRT5 in H295R cells grown under normal conditions (GM), starvation (SM), metformin (Met) (1mM) treatment and resveratrol (RSV) (5μM) treatment. SIRT3 was significantly upregulated after 3 hours of resveratrol treatment and after 24 hours of metformin treatment. SIRT1 and SIRT5 expression seemed not to be altered. Data are shown as the mean ± SD of three independent experiments. * p<0.05, ** p<0.01. B. Protein expression of SIRT1, SIRT3 and SIRT5 in H295R cells grown under different conditions assessed by Western blot analyses. Effects were assessed on endogenous SIRTs as well as on transfected SIRTs (after transfecting cells with SIRT1-, SIRT3- or SIRT5-pcDNA3 constructs). Representative Western blots are shown. Neither starvation nor metformin or resveratrol treatment changed the SIRT1, SIRT3 or SIRT5 protein expression. Expression of SIRT3 was not detectable in non-transfected cells. β-actin staining was used as loading control.

Resveratrol is reported to promote gene expression of *SIRT1* and thereby to enhanceprogesterone production of ovarian granulosa cells [21]. Moreover, resveratrol is also reported to enhance cortisol biosynthesis by a SIRT3 and SIRT5 dependent mechanism [22]. Therefore, to assess whether resveratrol may act through SIRT1/3/5 to modulate steroidogenesis in H295R cells, we assessed mRNA expression of *SIRT1/3/5* after resveratrol treatment for 3 to 24 hours (Fig 3A). Gene expression profiling using specific qRT-PCR showed no changes after 6 and 24 hours of resveratrol treatment. However, short term resveratrol treatment of 3 hours showed a significantly higher expression of SIRT3 (Fig 3A). Similarly, we found an upregulation of *SIRT3* mRNA after 24 hours of metformin treatment. Starvation had no effect on *SIRT1/3/5* expression, which is in line with work from our lab studying gene expression profiles in starved H296R cells using the Affymetrix gene chip [27].

We also performed Western blot experiments to assess an effect of resveratrol on sirtuins at the protein level. This was tested on endogenous sirtuins in H295R cells, as well as after transiently overexpressing SIRT1/3/5 in the cells. However, we did not find any alterations on protein expression of SIRT1/3/5 in H295R cells after resveratrol treatment compared to controls (Fig 3B). This indicates that the effect of resveratrol is most likely not mediated through sirtuins.

### Effect of sirtuins on steroidogenesis

**Fig 4:**
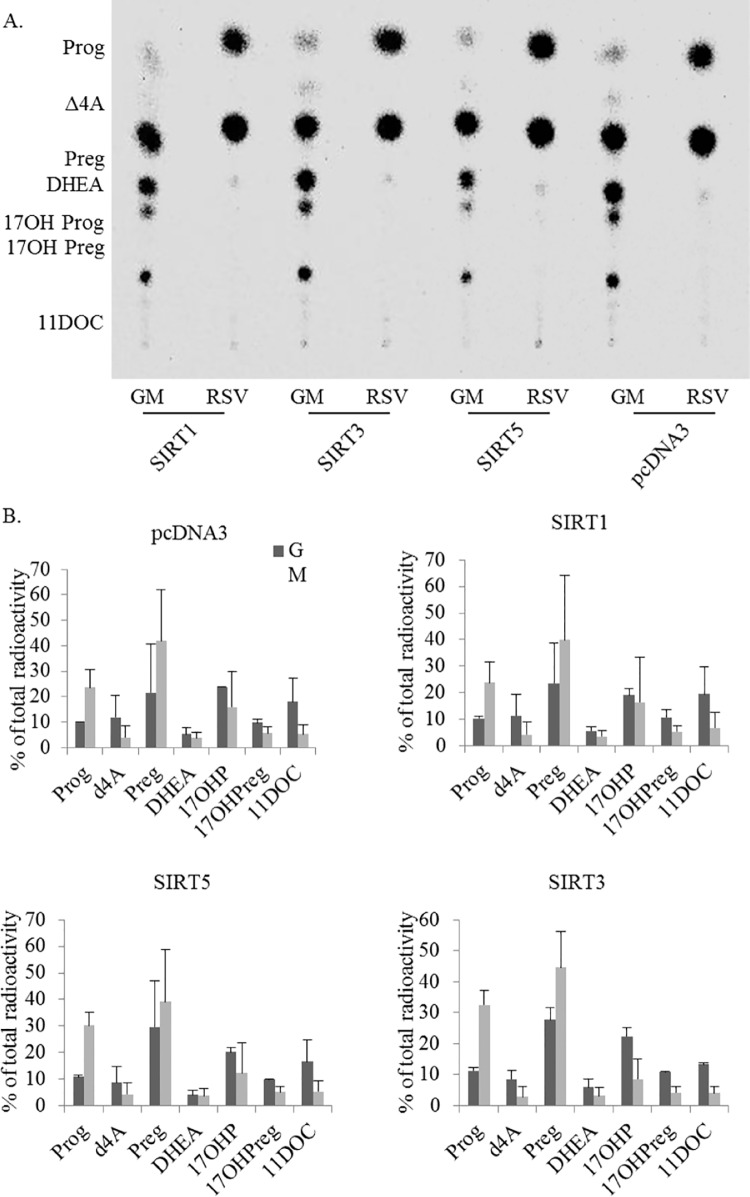
Overexpression of sirtuins has no effect on steroidogenesis in H295R cells. H295R cells were transfected with human SIRT1, SIRT3 and SIRT5, respectively and grown under normal conditions (GM) or under resveratrol (RSV) (5μM) treatment. Transfection with empty vector pcDNA3 was used as a control. A. Representative steroid profile obtained from transfected H295R cells. Steroid production was labeled with [3H]-pregnenolone for 90min and extracted steroids were resolved by TLC. B. Quantitative analysis of steroid production showed no alterations after overexpression of sirtuins. Data are indicated as the mean ± SD of three independent experiments. TLC, thin layer chromatography; Prog, progesterone; Δ4A, androstenedione; Preg, pregnenolone; 17OHPreg, 17-hydroxypregnenolone; 17OHP, 17-hydroxyprogestertone, 11DOC, 11-deoxycortisol.

Because *SIRT3* expression was enhanced after 3 hours of resveratrol treatment (Fig 3A), the direct effect of *SIRT1*, *3* and *5* on adrenal steroidogenesis was tested. For that, we overexpressed human SIRT1, SIRT3 and SIRT5 in H295R cells. To further assess whether different growth conditions influence a sirtuin-dependent regulation of steroidogenesis, we also overexpressed SIRT1/3/5 in starved and resveratrol treated cells. We then compared the steroid profiles of the human adrenal cells overexpressing SIRT1, SIRT3, and SIRT5 to the steroid profile of control cells in all conditions. Transfection was controlled by assessing overexpression of specific proteins (Western blot analyses, data not shown). In summary, all these experiments revealed no effect of sirtuins on steroidogenesis of H295R cells (Fig 4). Furthermore, we also show that sirtuin overexpression does not alter the effect of starvation or resveratrol treatment on androgen production. Therefore, resveratrol seems to act on the steroidogenesis of H295R cells in a sirtuin-independent manner.

### Resveratrol effect on the PKB/Akt pathway in H295R cells

**Fig 5:**
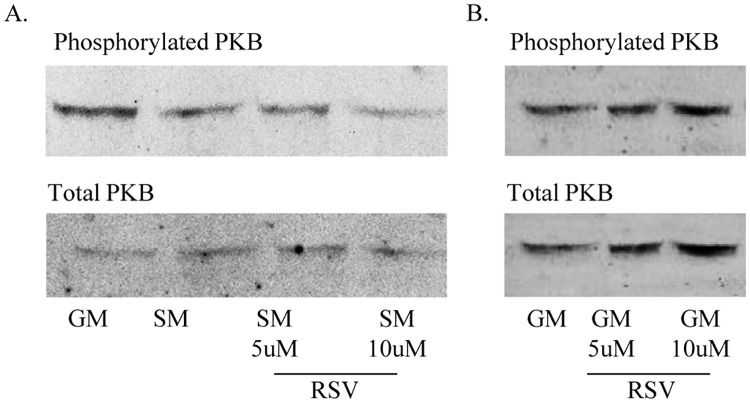
Resveratrol does not alter CYP17 and CYP21 expression via PKB signaling. H295R cells were treated with 5μM or 10μM resveratrol (RSV) for 6h. Total and phosphorylated PKB protein expression was assessed by Western blot analysis. A. H295R cells grown under starvation (SM) showed a dose-dependent downregulation of PKB phosphorylation after RSV treatment. B. In H295R cells grown under normal growth (GM) condition no effect of RSV on PKB phosphorylation was observed.

Resveratrol is reported to inhibit steroidogenesis through the PKB/Akt signaling pathway in rat ovarian Theca cells [13]. This pathway is also inhibited by starvation growth conditions that change steroidogenesis in adrenal H295R cells [25]. Therefore, we hypothesized that resveratrol may act via PKB/Akt signaling. Using Western blot experiments we found a resveratrol dependent downregulation of Akt-phosphorylation in cells grown under starvation growth conditions (Fig 5A). Cells cultured under starvation showed a dose-dependent inhibition of PKB signaling after 30 minutes resveratrol treatment compared to starvation control conditions, while in normal growth media we observed higher PKB signaling activity after 30 minutes (Fig 5A). By contrast, this inhibitory effect of resveratrol on PKB signaling was not found in H295R cells grown under non-starved conditions (Fig 5B). However, as resveratrol also lowers androgens in non-starved cells (Fig 1), this indicates that resveratrol may not only act through the PKB signaling pathway, but uses other (as yet unidentified) mechanisms for its action.

### Resveratrol and CYP17, CYP21 protein degradation

**Fig 6:**
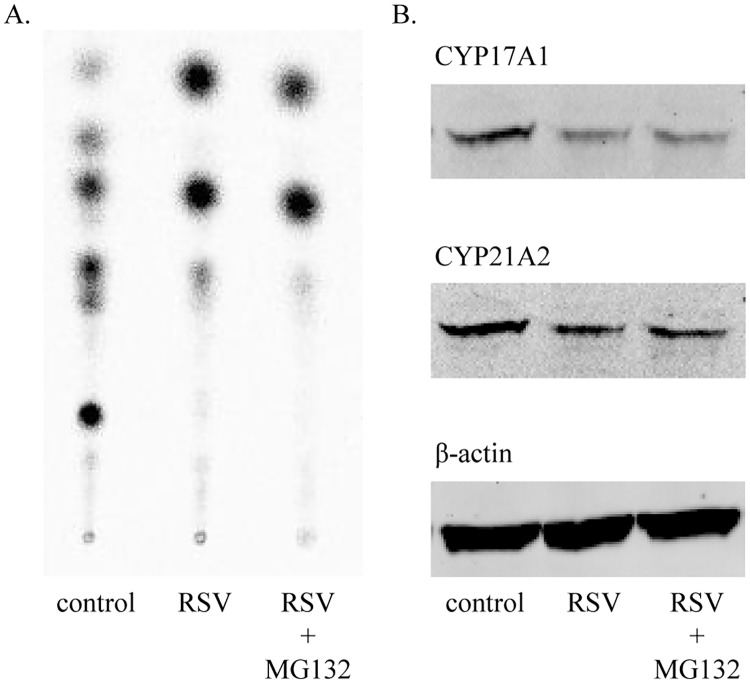
Inhibition of protein degradation does not rescue CYP17 and CYP21 protein expression and activity. H295R cells were treated with 5μM resveratrol (RSV) for 6h in the presence and absence of a protein degradation inhibitor (MG132). A. Steroid profile (TLC) showing no effect of the protein degradation inhibitor MG132 on steroid conversion. B. Western blot showing no effect of MG132 on CYP17 and CYP21 protein expression under RSV treatment.

Because the effect of resveratrol on H295R cell steroidogenesis was found after 6 hours, and because we found no changes at the RNA level, but specific changes at the protein level, we hypothesized that resveratrol might promote protein degradation. We therefore performed resveratrol treatment experiments under the effect of the protein degradation inhibitor MG132. However, MG132 was not able to reverse the effect of resveratrol on CYP17 and CYP21 protein expression and activities (Fig 6). Therefore, resveratrol is unlikely to act on proteasomal degradation.

## Discussion

A recent clinical study in PCOS women showed that RSV reduces androgens produced at too high amounts by the ovaries and the adrenals by more than 20% [10]. But the exact mechanism of action of RSV is unknown. In this study, we confirm that resveratrol inhibits steroidogenesis in adrenocortical H295R cells, reflecting the treatment effect of RSV seen in humans in the cell model. Steroid biosynthesis is complex and involves multiple different enzymes and cofactors, which are regulated at several levels [28].

To investigate where resveratrol acts exactly on steroid biosynthesis, we studied critically involved enzymes at the RNA and protein expression level, and also on the functional level. In line with reported studies, we found lower enzyme activities for CYP17 and CYP21 under resveratrol treatment [15, 16, 29]. Investigating the underlying mechanism, we found correspondingly lower CYP17 and CYP21 protein expressions, but no alterations at the mRNA expression level. These findings indicated that RSV might not affect gene transcription or RNA stability, but rather posttranslational events such as protein degradation. Using MG132, we also excluded an effect of RSV on proteasomal protein degradation.

We therefore studied other possible modulators and signaling pathways used by resveratrol regulating steroidogenesis. The family of sirtuins comprises known RSV targeted regulators of steroidogenesis such as StAR cholesterol transporter [21] and CYP11A1 enzyme activity [22]. RSV has been reported to stimulate cortisol synthesis by SIRT3-dependent deacetylation of CYP11A1 in H295R cells [22]. Although we could show that resveratrol alters mRNA expression of *SIRT3*, no change of SIRT3 was found at the protein level. Moreover we did not find changes of mRNA or protein expression of SIRT5 in RSV treated H295R cells. In addition, SIRT1 expression was also not altered by RSV in our H295R cell experiments, which is in contrast to a reported finding in rat ovarian granulosa cells [21]. Thus RSV effects may be cell-, tissue- or even species-specific. Other than previous studies, we also assessed the direct effect of sirtuins on steroidogenesis. Overexpression of sirtuins 1, 3 and 5 did not alter the steroid profile of H295R cells. Thus, sirtuins seem not to be involved in RSV modulating androgen production in H295R cells.

In rat ovarian theca-interstitial cells the effect of resveratrol on steroidogenesis seems mediated through an inhibition of PKB/Akt signaling [13]. Similarly, the effect of starvation on androgen production involves PKB/Akt signaling [25]. We now show that RSV also inhibits PKB/Akt signaling of H295R cells, but only under starvation growth conditions. As RSV also inhibits androgen production of H295R cells grown under normal growth conditions, additional targets of RSVneed to be elucidated.

In conclusion, this study shows that RSV inhibits steroidogenesis in human adrenocortical cells by lowering protein expression and inhibiting enzyme activities of CYP17 and CYP21. This effect is not mediated by sirtuins and not regulated by proteasomal degradation. PKB/Akt signaling seems to be involved. These properties of action together with recent clinical findings make RSV a candidate for the treatment of hyperandrogenic disorders such as PCOS.

## Supporting information

S1 Fig Original pictures of Western blots found throughout the paper. Original Western blots to Fig 2. Representative captures are given in Fig 2B.
(PDF)

S1 Table Sequences of all primers used in the study. (DOCX)
