# Supplementary material for: Resveratrol inhibits androgen production of human adrenocortical H295R cells by lowering CYP17 and CYP21 expression and activities
Source: PLoS One. 2017 Mar 21;12(3):e0174224. doi: 10.1371/journal.pone.0174224 (PMC5360261; doi:10.1371/journal.pone.0174224)

S1. Original pictures of Western blots found throughout the paper.

Original Western blots to Figure 2. Representative captures are given in Figure 2B.

2B.

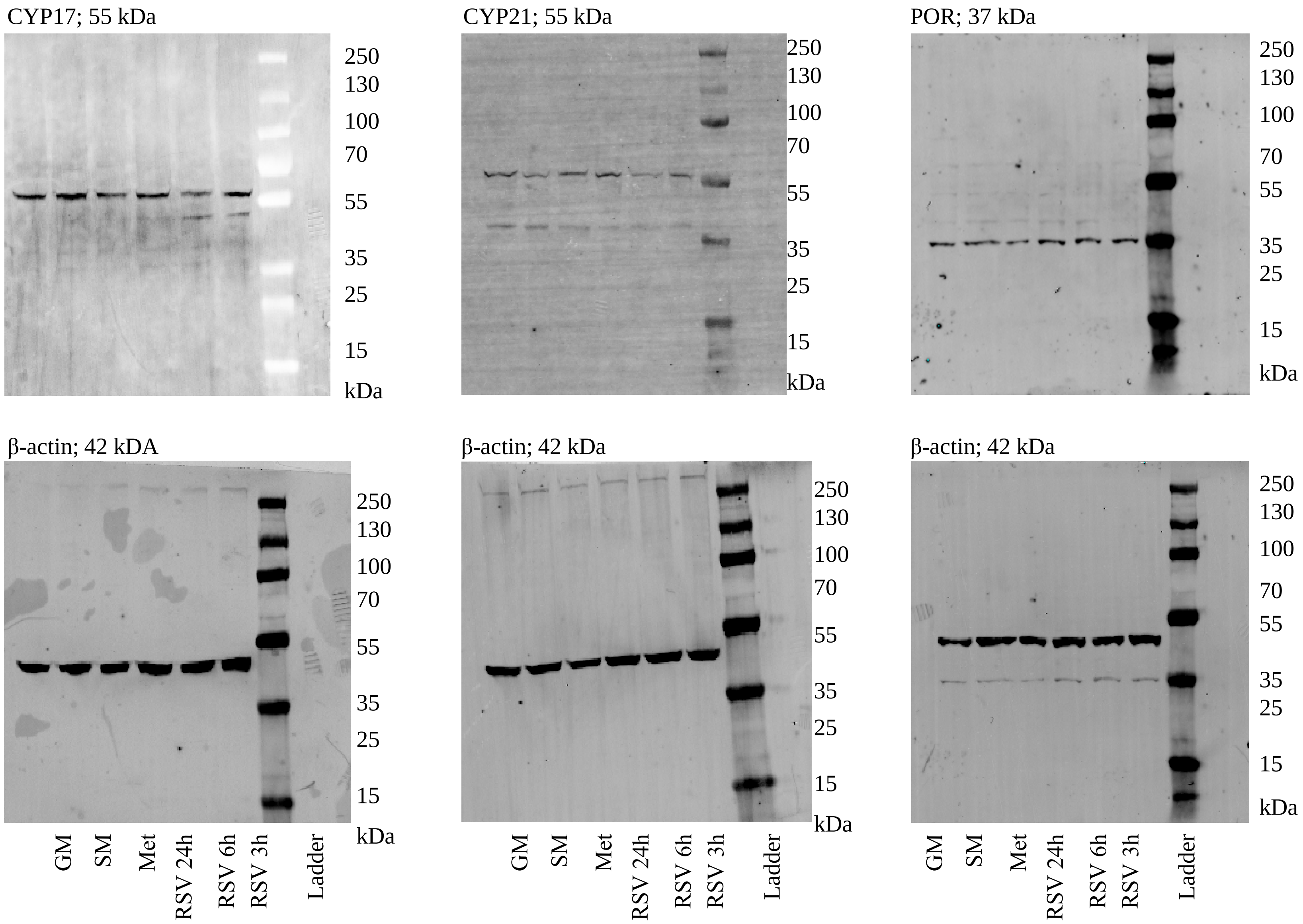

Original Western blots to Figure 3. Representative captures are given in Figure 3B.

3B.

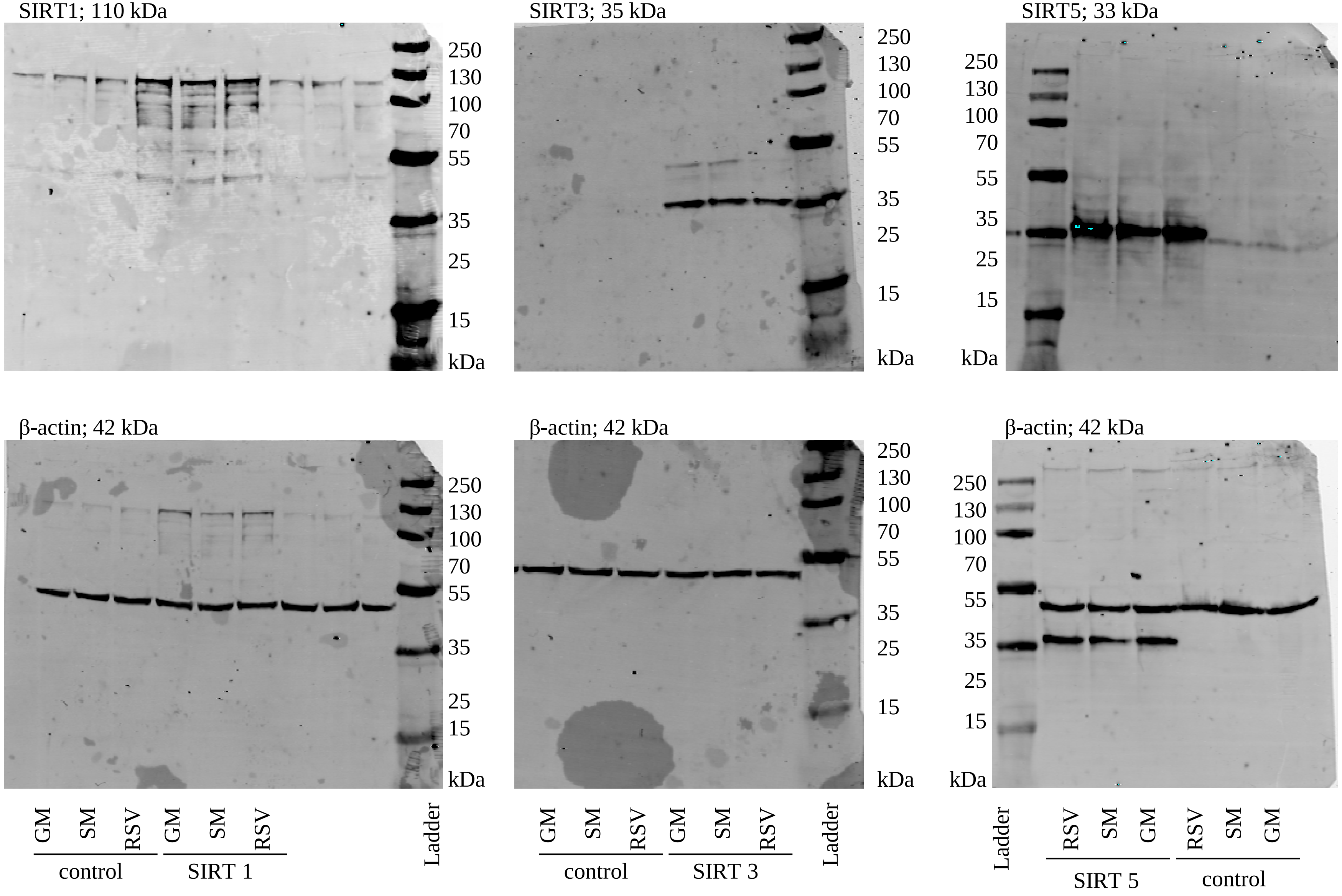

Original Western blots to Figure 5. Representative captures are given in Figure 5A and 5B.

5A. Phosphorylated PKB; 60

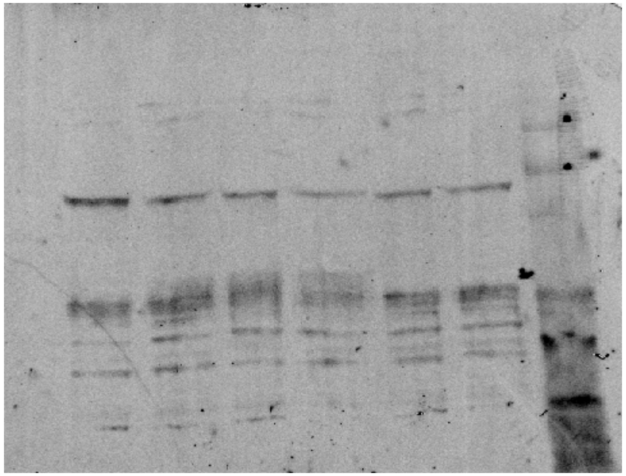

Total PKB; 60 kDa

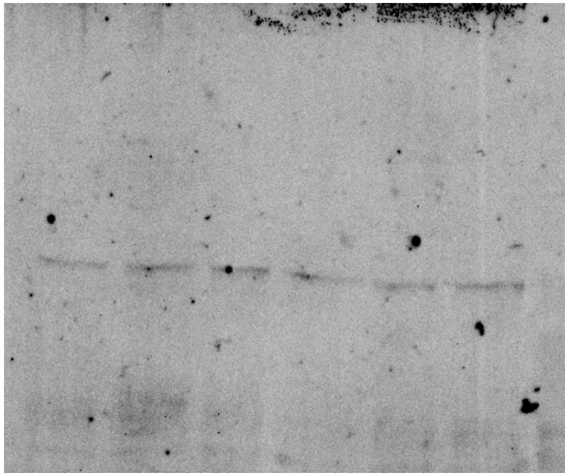

GM SM GM 5uM GM 10uM

RSV Metformin Ladder

5B. Phosphorylated PKB; 60 kDa

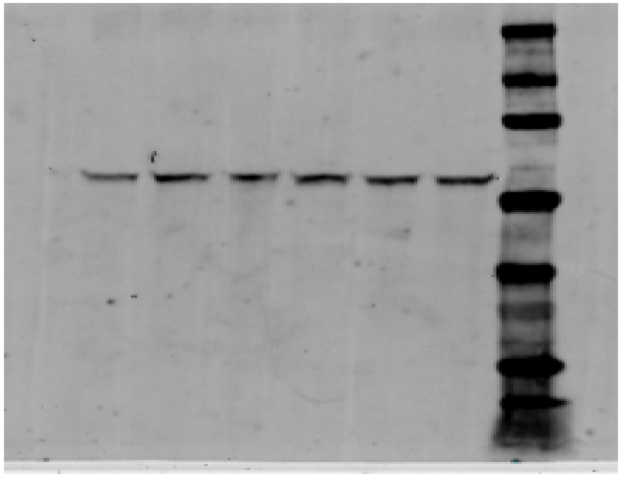

Total PKB; 60 kDa

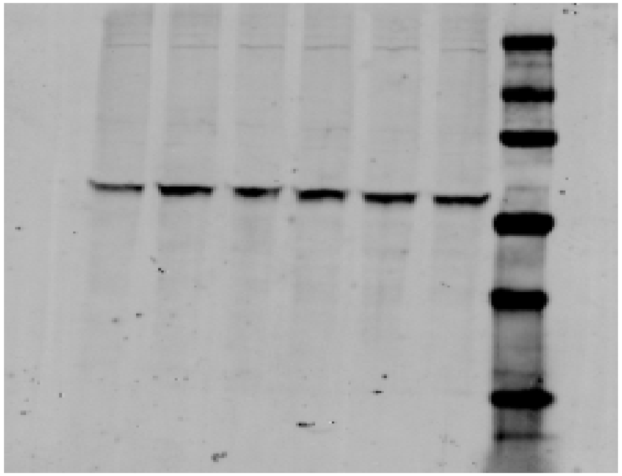

GM GM GM 5uM GM 10uM

RSV Metformin Ladder

Original Western blots to Figure 6. Representative captures are given in Figure 6.

6. CYP17A1; 55 kDA

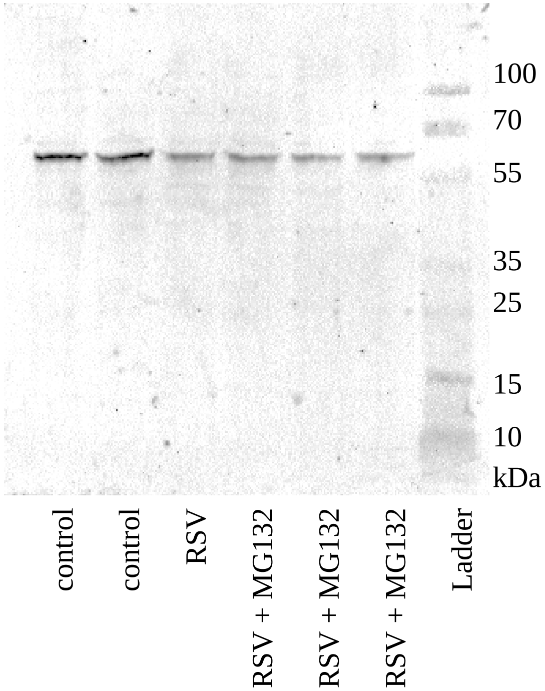

CYP21A2; 56 kDa

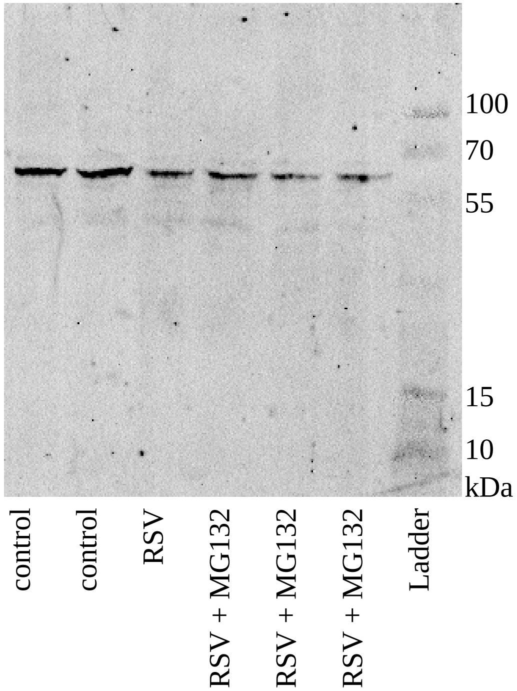

β-actin; 42 kDa

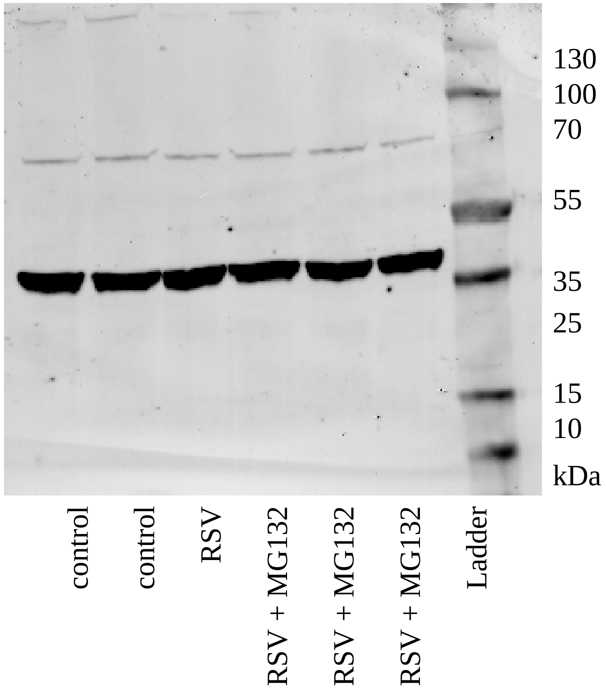

Supplement: S1 Fig — Original Western blots to Fig 2. Representative captures are given in Fig 2B. (PDF) [file pone.0174224.s001.pdf]
