# Supplementary material for: Resveratrol inhibits androgen production of human adrenocortical H295R cells by lowering CYP17 and CYP21 expression and activities
Source: PLoS One. 2017 Mar 21;12(3):e0174224. doi: 10.1371/journal.pone.0174224 (PMC5360261; doi:10.1371/journal.pone.0174224)
Supplement: S1 Table — (DOCX) [file pone.0174224.s002.docx]

**S1 Table. Sequences of all primers used in the study.**

| **Gene** | **Forward Primer** | **Reverse Primer** |
| --- | --- | --- |
| CYP17 | 5’-AGC CGC ACA CCA ACT ATC AG-3’ | 5’-TCA CCG ATG CTG GAG TCA AC-3’ |
| CYP21 | 5’-CTG AGC CAC TTA CCT ACA AGC-3’ | 5’-GAG CGG GTG AGC TTC TTG TG-3’ |
| POR | 5’-ACG ACG ATG GGA ACT TGG AG-3’ | 5’-TGG ACC ACA AGC TCG TAC TG-3’ |
| SIRT 1 | 5’-TAT GCT CGC CTT GCT GTA GAC-3’ | 5’-GTG ACA GAG AGA TGG CTG GAA T-3’ |
| SIRT 3 | 5’-CAG CAG CTC CCA GTT TCT TC-3’ | 5’-TCA GCT CAG CTA CAT CCT GC-3’ |
| SIRT 5 | 5’-GCG TCC ACA CGA AAC CAG AT-3’ | 5’-CGG AAC ACC ACT TTC TGC AC-3’ |
| GAPDH | 5’-TCT CCT CTG ACT TCA ACA-3’ | 5’-CCC TGT TGC TGT AGC CAA-3’ |
